# Teledermatology Within Correctional Settings in the United States: A Narrative Review of the Literature

**DOI:** 10.2196/47115

**Published:** 2023-05-26

**Authors:** Samir Kamat, Aneesh Agarwal, Timothy Klufas, Saahil Patel, Jun Lu

**Affiliations:** 1 Icahn School of Medicine at Mount Sinai New York City, NJ United States; 2 New York Medical College Valhalla, NY United States; 3 The College of New Jersey Ewing, NJ United States; 4 Department of Dermatology University of Conneticut Farmington, CT United States

**Keywords:** legal, patients who are incarcerated, vulnerable populations, teledermatology, volunteerism, correctional, teleconsultation, telemedicine, eHealth, skin disorders

Teledermatology is an emerging modality of care delivery. To broadly understand the role of teledermatology in the US correctional system, we conducted a narrative review using PubMed, Scopus, Embase, and gray literature. We identified 5 studies (Figure 1) analyzing over 1261 teledermatology encounters within correctional settings in the United States (summary characteristics are in Table 1; the search strategy used is in Multimedia Appendix 1).

The first published study on the use of teledermatology for incarcerated populations was in 1996 from East Carolina University in Greenville, North Carolina [1]. Since then, several single-center observational and cohort studies have reported the implementation of teledermatology across several localities, including Utah and Connecticut [2-4]. All studies have indicated the partnership between the dermatology providers and the state prison system. The Federal Bureau of Prisons (BOP) also established a teledermatology program in 2012 covering over 50 institutions. The collaboration between dermatologists and a government agency is critical and unique for teledermatology in correctional settings [5].

Teledermatology has proven to improve access to care and efficiently diagnose a broad spectrum of skin disorders, particularly inflammatory conditions, and skin infections. Common diagnoses reported included cutaneous infection [4], acne (9%-14.9%) [2-4], eczema (9.3%-18%) [2-4], psoriasis (28.1%) [3-4], and prurigo nodularis or lichen simplex chronicus (10%) [2]. One study showed that 86.3% of cases could be managed via teledermatology alone, with 86% of patients prescribed new topical therapeutics and 57.9% receiving systemic therapies, including biologics [4]. Medical management via teledermatology was confirmed to be successful and continued to serve patients well according to medical records [4]. When compared with face-to-face visit cohorts, teledermatology cohorts involved more medication recommendations (84.8% vs 48.4%; *P*<.001) and fewer procedures and referrals (*P*<.001), likely resulting from appropriate triage by a prison primary care physician [3].

Different teledermatology modalities have been adopted. Live videoconference is the most commonly implemented modality via various videoconference platforms, including Picture Tel, Skype, Zoom, etc. Store-and-forward has also been used alone or in combination with live video teledermatology (Table 1). Due to a lack of private internet access for inmates, all teledermatology encounters were conducted via institution health care staff, the provider-to-provider module. Teledermatology and face-to-face encounters can be transitioned both ways. Patients who need procedures or biopsies for diagnosis often require face-to-face visits but may transfer back to teledermatology for continuous care after surgery or a definite diagnosis [3,4].

In addition to improved access, teledermatology in one program decreased wait time with an average turnover time of 1-2 weeks compared with 4-12 weeks for an in-person consultation [5]. The economic benefits are significant. According to the BOP report, there is an average of US $895 in savings per teledermatology consult from administration costs, particularly regarding securing transportation [5].

Overall, patients who are incarcerated are an underserved population with limited access to specialty care. Teledermatology has increased access and shown capability in addressing wide-spectrum conditions with economic benefits. Future teledermatology initiatives in correctional settings may prioritize high-quality photographs with video, integrate teledermoscopy to aid in diagnosing, emphasize the continuity of care, and expand to more sites.

**Figure 1:**
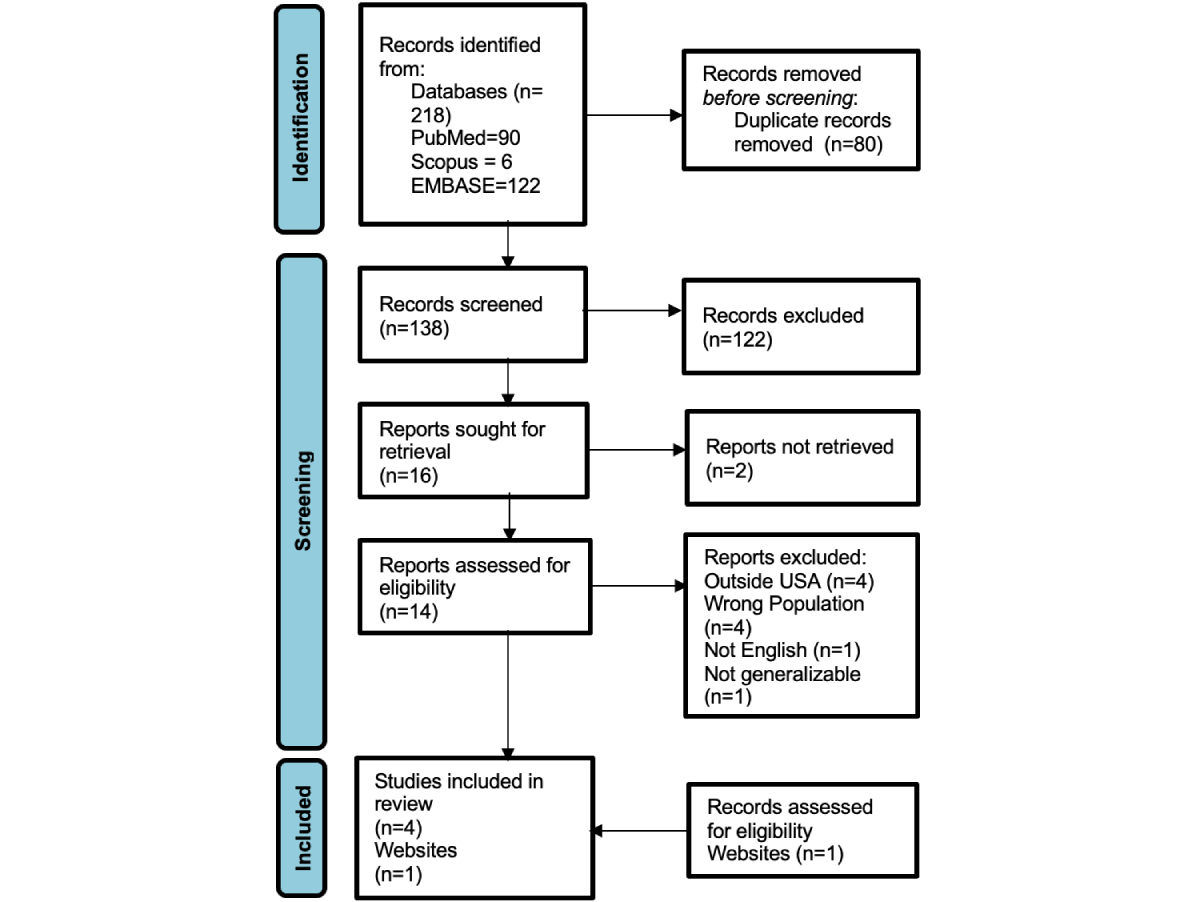
PRISMA (Preferred Reporting Items for Systematic Reviews and Meta-Analyses): teledermatology in correctional settings.

**Table 1:** Studies reporting on the use of teledermatology in correctional settings.

Study	Population/sample	Type of consultation	Results	Conclusion
Norton et al [1], 1997	189 teleconsultations	Live video (REACH-TV)	Most common diagnosis included: eczema, appendageal disorders, papulosquamous disorders Cost saving of US $1000 per visit 355 specific treatment recommendations 66 diagnostic recommendations	Remote visits yielded monetary and time savings compared to resources needed for face-to-face visits
Phillips et al [2], 1996	138 teleconsultations	Live video (Picture Tel 4000)	159 diagnoses and 252 treatments Eczema and acne common diagnosis 72% African American/average age 32 years	Provider confidence in diagnostic capabilities and ability to successfully manage patient care
Clark et al [3], 2021	779 encounters from 359 patients (335 teleconsultations, 444 face-to-face)	Live video vs face-to-face	Psoriasis (28.1%), acne (14.9%), unspecified rash (9.3%) Teledermatology less likely led to secondary diagnosis (52% vs 26.3%; *P*<.001) Teledermatology more likely to prescribe medication (84.8% vs 48.4%; *P*<.001) but less likely to get referred for procedures (*P*<.001) The average teledermatology follow-up period was 2.3 months vs 4.8 months for face-to-face visits (*P*<.001)	Cost-effective for managing common skin conditions. Success with managing severe psoriasis and acne even when using systemic treatments and lab monitoring.
Stoj and Lu [4], 2021	98 teleconsultations	Live video (Skype) and store-and-forward	Teledermatology diagnoses: 78.1% (57/73) new diagnoses, and 17 consistent with established diagnoses 86.3% (63/73) diagnoses involved only telemedicine after initial diagnosis Face-to-face was required for 21.9% (16/73) and 13/16 being subsequently managed with telemedicine	Effective for diagnosing and managing acute and chronic dermatological conditions including those that require systemic treatment
Federal Bureau of Prisons (website) [5], 2014	Per 2014, 50+ institutions across the Bureau of Prisons, 501 consults in 2013	Store-and-forward	US $448,395 annual savings Teledermatology consultation wait time 1-2 weeks in correctional setting vs 30-90 days in correctional setting Identifying optimal medications via efficacy and costs considerations Average saving of US $895 per visit	Significant savings, reduction in wait times, continuity of care, and expanded reach to geographically inaccessible or rural areas

## Appendix

Multimedia Appendix 1 Search strategy.

## Abbreviations

BOP: Bureau of Prisons

## References

[1] Norton SA, Burdick AE, Phillips CM, Berman B (1997). Teledermatology and underserved populations. Arch Dermatol.

[2] Phillips CM, Murphy R, Burke WA, Laing VB, Jones BE, Balch D, Gustke S (1996). Dermatology teleconsultations to Central Prison: experience at East Carolina University. Telemed J.

[3] Clark JJ, Snyder AM, Sreekantaswamy SA, Petersen MJ, Lewis BK, Secrest AM, Florell SR (2021). Dermatologic care of incarcerated patients: a single-center descriptive study of teledermatology and face-to-face encounters. J Am Acad Dermatol.

[4] Stoj V, Lu J (2021). Comment on: "The growth of teledermatology: Expanding to reach the underserved". J Am Acad Dermatol.

[5] Teledermatology program improves dermatologic care. Federal Bureau of Prisons.

