# Supplementary material for: Teledermatology Within Correctional Settings in the United States: A Narrative Review of the Literature
Source: JMIR Dermatol. 2023 May 26;6:e47115. doi: 10.2196/47115 (PMC10335332; doi:10.2196/47115)
Supplement: Multimedia Appendix 1 [file derma_v6i1e47115_app1.docx]

**Search Strategy for Individual Academic Databases**:

The narrative review focused on answering the question “What are the characteristics of teledermatology implementations in correctional settings across the United States” through searches in Pubmed, Scopus, Embase, and Google (grey literature). The condition/domain of focus was teledermatology for any skin condition in correctional settings. The participant/population of interests included incarcerated patients within the United States. Interventions/Exposures included teledermatology initiatives, including video teleconferencing, store-and-forward, hybrid, phone calls, and digital health. The main outcomes of interest included the number of teleconsultations/diagnoses, modality of teledermatology, patient demographics, cost savings, rates of follow-up, and key takeaways. Two reviewers screened articles according to title and abstract and reviewed full text articles before arriving at a final selection of articles for the data extraction. The inclusion criteria were (1) include incarcerated patients (2) published in English, (3) include quantitative data, (4) include teledermatology (5) discuss any of the following: a) conditions treated b) diagnosis and management c) clinician and patient satisfaction d) follow-up after telemedicine encounters e) logistical implementation of teledermatology f) within the United States g) cost and access. Covidence, an automated screening and extraction tool, was used to facilitate execution of the search strategy.”

**Search strategy for MEDLINE-Ovid**

| 1 | Dermatology/ | 20806 |
| --- | --- | --- |
| 2 | exp Skin Diseases/ | 1132033 |
| 3 | Telemedicine/ | 35781 |
| 4 | 1 or 2 | 1143673 |
| 5 | 3 and 4 | 1331 |
| 6 | (Telederm* or Tele-derm*).mp. | 1348 |
| 7 | (Dermatol* adj3 (telemedicine or tele-medicine or telehealth or tele-health or teleconsult* or tele-consult* or Store-and-forward or electronic consult* or econsult* or e-consult*)).mp. | 148 |
| 8 | 5 or 6 or 7 | 2016 |
| 9 | exp Correctional Facilities/ | 11309 |
| 10 | (prison* or jail* or correctional).mp. | 35091 |
| 11 | Prisoners/ | 18189 |
| 12 | (Imprison* or Inmate* or Incarcerate* or underserve*).mp. | 34401 |
| 13 | 9 or 10 or 11 or 12 | 60044 |
| 14 | 8 and 13 | 90 |

**Search strategy for EMBASE-Ovid**

| 1 | Dermatology/ | 54041 |
| --- | --- | --- |
| 2 | exp Skin Disease/ | 1903048 |
| 3 | Telemedicine/ | 41081 |
| 4 | 1 or 2 | 1923072 |
| 5 | 3 and 4 | 1454 |
| 6 | teledermatology/ | 1584 |
| 7 | (Telederm* or Tele-derm*).mp. | 2297 |
| 8 | (Dermatol* adj3 (telemedicine or tele-medicine or telehealth or tele-health or teleconsult* or tele-consult* or Store-and-forward or electronic consult* or econsult* or e-consult*)).mp. | 187 |
| 9 | 5 or 6 or 7 or 8 | 3393 |
| 10 | exp Correctional Facility/ | 2621 |
| 11 | (prison* or jail* or correctional).mp. | 42915 |
| 12 | Prisoner/ | 19762 |
| 13 | (Imprison* or Inmate* or Incarcerate* or underserve*).mp. | 39876 |
| 14 | 10 or 11 or 12 or 13 | 71855 |
| 15 | 9 and 14 | 122 |

**Search strategy for SCOPUS:**

6 results

( TITLE-ABS-KEY ( telederm* OR tele-derm* ) AND TITLE-ABS-KEY ( dermatol* W/3 (telemedicine OR tele-medicine OR telehealth OR tele-health OR teleconsult* OR tele-consult* OR store-and-forward OR electronic AND consult* OR econsult* OR e-consult*) ) AND TITLE-ABS-KEY ( prison* OR jail* OR correctional OR imprison* OR inmate* OR incarcerate* OR underserve* ) )

**Search Strategy for Grey Literature**:

**Search Strategy for Google Search Engine:**

telederm* | tele-derm* prison* | jail* | correctional | imprison* | inmate* | incarcerate* | underserve*
